# Dynamics of degeneration and regeneration in developing zebrafish peripheral axons reveals a requirement for extrinsic cell types

**DOI:** 10.1186/1749-8104-7-19

**Published:** 2012-06-08

**Authors:** Rosario Villegas, Seanna M Martin, Kelley C O’Donnell, Simon A Carrillo, Alvaro Sagasti, Miguel L Allende

**Affiliations:** 1 FONDAP Center for Genome Regulation, Facultad de Ciencias, Universidad de Chile, Las Palmeras 3425, Santiago, Chile; 2 Department of Molecular Cell and Developmental Biology, University of California, Los Angeles, CA 90095, USA

**Keywords:** In vivo axotomy, Wallerian degeneration, Schwann cells, Leukocytes, Hair cells, Neurons, Lateral line

## Abstract

**Background:**

Understanding the cellular mechanisms regulating axon degeneration and regeneration is crucial for developing treatments for nerve injury and neurodegenerative disease. In neurons, axon degeneration is distinct from cell body death and often precedes or is associated with the onset of disease symptoms. In the peripheral nervous system of both vertebrates and invertebrates, after degeneration of detached fragments, axons can often regenerate to restore function. Many studies of axonal degeneration and regeneration have used in vitro approaches, but the influence of extrinsic cell types on these processes can only be fully addressed in live animals. Because of its simplicity and superficial location, the larval zebrafish posterior lateral line (pLL) nerve is an ideal model system for live studies of axon degeneration and regeneration.

**Results:**

We used laser axotomy and time-lapse imaging of pLL axons to characterize the roles of leukocytes, Schwann cells and target sensory hair cells in axon degeneration and regeneration in vivo. Immune cells were essential for efficient removal of axonal debris after axotomy. Schwann cells were required for proper fasciculation and pathfinding of regenerating axons to their target cells. Intact target hair cells were not themselves required for regeneration, but chemical ablation of neuromasts caused axons to transiently deviate from their normal paths.

**Conclusions:**

Macrophages, Schwann cells, and target sensory organs are required for distinct aspects of pLL axon degeneration or regeneration in the zebrafish larva. Our work introduces a powerful vertebrate model for analyzing axonal degeneration and regeneration in the living animal and elucidating the role of extrinsic cell types in these processes.

## Background

Axonal degeneration occurs during normal development of the nervous system and is central to the pathology of neurodegenerative diseases, nerve damage caused by metabolic diseases, and mechanical nerve injuries [1-3]. While there are different types of axonal degeneration, similar mechanisms regulate both developmental pruning of excessive axonal branches and the selective removal of damaged axons [1,4,5]. Wallerian degeneration (WD) occurs in axon fragments that are separated from their cell body [6]. WD occurs in a sterotyped and orderly fashion, implying that it is under genetic control, and has been described in the central and peripheral nervous system after trauma, stroke or infection [1,3]. Immediately after an axon is severed, acute axonal degeneration (AAD) can occur at both ends adjacent to the cut [7]. Following AAD, the detached axon fragment remains intact during a characteristic “lag phase”. Following the lag phase, axons quickly degenerate: the endoplasmic reticulum breaks down, neurofilaments degrade, mitochondria swell and the axon fragments [1]. Finally, in the last phase of WD, axon fragments are removed by phagocytic cells.

In the peripheral nervous system (PNS), Schwann cells and macrophages play important roles throughout the process of WD. Schwann cells decrease the synthesis of myelin lipids during the first 12 hours after axotomy [8] and stop producing myelin proteins within 48 hours [9]. In the absence of macrophages, the process is even more rapid [3,10], with glial cells removing myelin during the earliest stages of PNS axon degeneration [11-14]. Glial cells also release chemokines and cytokines, some of which are responsible for recruiting macrophages to the site of nerve degeneration in the final phase of myelin removal [15,16]. After injury, these cells adhere to the basal lamina, enter the nerve, and phagocytose opsonized debris [17-20].

The limited regenerative capacity of neurons in the adult central nervous system (CNS) of mammals has been a subject of intense study. Several studies in vertebrate models have established that, in addition to intrinsic growth programs, extrinsic factors regulate axonal regeneration [21,22]. For example, inhibitory molecules associated with myelin and glial scars are induced by axotomy and create obstacles to axonal regeneration in the CNS. Astrocytes, which form glial scars in the CNS after injury, are absent in the peripheral nervous system (PNS), though inhibitory molecules associated with myelin are expressed in the PNS [23]. However, in the PNS, Schwann cells undergo dedifferentiation after injury, diminishing the effect of inhibitory proteins [24]. Schwann cells and macrophages in the PNS can have positive effects on regeneration by rapidly removing myelin debris [25] and expressing a wide range of neurotrophic factors that create a favorable environment for axonal growth [22]. Glia can also serve as guides by providing structural substrates to facilitate the growth of axons along their original paths [26].

The zebrafish lateral line (LL) has been a useful model for understanding interactions between axons and extrinsic cell types, including glial cells [27], during development, but it has yet to be exploited for studies of axon degeneration and regeneration. The LL is a mechanosensory system that responds to mechanical stimuli produced by water movement [28]. It is composed of groups of individual sensory organs called neuromasts, which are distributed on the body surface in species-specific patterns. Each neuromast contains 15 to 20 hair cells at its core, surrounded by two types of accessory cells: supporting cells and mantle cells. Hair cells are innervated by afferent sensory neurons whose cell bodies are located in either the anterior or posterior LL ganglia. LL neurons extend their central axons to the hindbrain [29-31] in a somatotopic fashion [32,33]. Approximately 20 bipolar afferent neurons receive synaptic input from hair cells of each major branch of the lateral line. Most studies to date have focused on the posterior LL (pLL), which extends along the trunk and tail.

To determine whether extrinsic cell types can influence axon degeneration or regeneration in the PNS, we have characterized these processes after pLL nerve axotomy*.* This approach allowed us to quantitatively describe the onset and progression of WD in axotomized pLL neurons and to follow their regeneration. We found that removal of glia, leukocytes, and target cells each had a distinct effect on different aspects of axon degeneration or regeneration. Together, these studies establish the zebrafish pLL nerve as a powerful model for live studies of axon degeneration and regeneration and uncover a rich variety of cell-cell interactions that regulate these processes.

## Results

### Lateral line axons undergo Wallerian degeneration after axotomy

The zebrafish posterior lateral line (pLL) is an excellent model for studying peripheral axonal structure and function *in vivo.* The pLL nerve is long and superficially located, target cells in neuromasts are located along the body surface in stereotyped positions, and all cell types in the system can be genetically, physically or chemically ablated. These properties made it possible for us to use laser axotomy and time-lapse imaging to monitor axon degeneration and regeneration after injury to lateral line axons in live zebrafish larvae [34]. To study the behavior of the entire nerve we used the *neuroD::EGFP* stable transgenic line, and to analyze the behavior of single neurons we injected the *HuC::GFP* transgene at the single cell stage and screened for transient transgenic embryos expressing GFP in single lateral line neurons at three days postfertilization (dpf). Thus, we were able to transect all axons in the nerve using the *NeuroD::EGFP* transgenic fish line, and sever single axons with *HuC::GFP* transient transgenics, presumably leaving the rest of the pLL nerve intact. Neurons were axotomized at 78 hours postfertilization (hpf) using a two-photon microscope and imaged at one- two- or twenty-minute intervals for up to twelve hours with confocal microscopy. We chose three dpf fish because at this stage the pLL system and innate immune leukocytes are functional. At three dpf Schwann cells have differentiated, overlie the pLL nerve, and express myelin, though the myelin sheath only forms later, between four and seven dpf [35,36].

**Figure 1:**
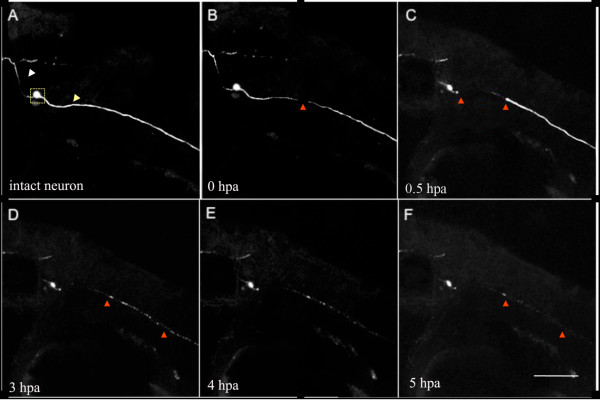
**Acute axonal degeneration (AAD) and Wallerian degeneration are observed in the zebrafish posterior lateral line (pLL) nerve after laser axotomy.** A three dpf larva, injected with *HuC::GFP* to image and axotomize a single pLL neuron. **(a)** Intact neuron with a typical bipolar morphology. White arrowhead shows central projection, yellow arrowhead shows peripheral projection, and square shows cell body. **(b)** Zero hours postaxotomy (hpa), arrowhead points to position of laser axotomy. **(c)** Thirty minutes after axotomy. AAD was complete: arrowheads indicate extent of AAD. **(d)** Three hours after axotomy, the distal axon began to fragment (arrowheads). **(e)** Four hours after axotomy, some fragments remained. **(f)** Five hours postaxotomy, clearance of debris was almost complete. Scale bar, 100 μm.

**Figure 2:**
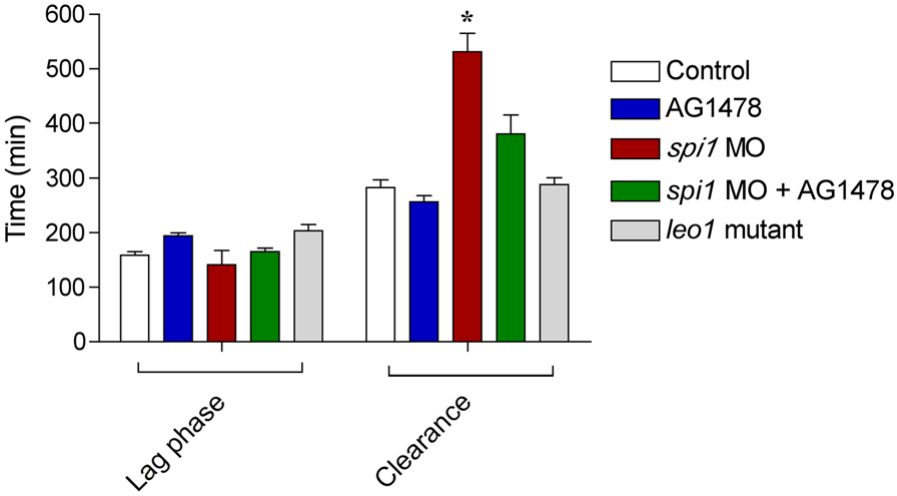
**Leukocytes and Schwann cells contribute to axon degeneration in the axotomized posterior lateral line nerve.** Length of lag phase (time from axotomy to fragmentation) and clearance phase (time from axotomy to axonal fragment removal) in minutes in control animals, animals lacking Schwann cells (AG1478 inhibitor and *leo1* mutants), animals lacking leukocytes (*spi1* MO) and animals lacking both cell types (*spi1* MO + AG1478). The absence of leukocytes, but not Schwann cells, significantly prolonged the clearance phase (n = 10); however, combined depletion of leukocytes and Schwann cells restored the normal time course of debris clearance. *Significant difference, two-way ANOVA, *P* <0.05.

After axotomy, disconnected axon fragments underwent three characteristic phases of Wallerian degeneration (WD): a lag phase (phase one), a fragmentation phase (phase two) and a clearance phase (phase three; Figure 1; see Additional File 1 for a time-lapse movie). The fragmentation and clearance phases began approximately three and five hours postaxotomy, respectively (Figure 2). WD of zebrafish pLL axons occurred much more rapidly than WD described in mammals and in *Drosophila*[37], but slightly more slowly than in the peripheral arbors of zebrafish trigeminal neurons [4]. In mice, prior to the onset of WD, dorsal root ganglion (DRG) neurons undergo acute axonal degeneration (AAD), generating a gap of 300 μm between adjacent ends of the axotomized nerve [9], but severed zebrafish trigeminal axons do not display AAD [4]. To determine whether AAD occurs in the pLL nerve, we imaged severed axons at one-minute intervals during the first hour postaxotomy in *NeuroD::EGFP* larvae. Similar to mouse DRG axons, a symmetrical dying back of both ends close to the site of LL axotomy occurred during this period, generating an approximate 150 μm gap at the injury site (see Figure 1 and movie in Additional File 2). 

To determine whether fragmentation occurs progressively as a wave from the lesion site to the axon terminal (proximal to distal), as has been described in other systems [7,38-40], synchronously along the entire axon, or distal to proximal [1], we imaged single severed axons every two minutes after axotomy at two different positions along the axon fragment: adjacent to the injury site and approximately 800 to 1000 μm further down the axon (Additional Files 3, 4 and 5). Imaging fragmentation at these two regions revealed that the most distal region fragmented before the more proximal region (n = 6; Additional Files 3 and 4). Imaging shorter (500 μm) fragments indicated that the timing and progression of fragmentation varied widely from axon to axon (Additional File 5). In some cases, fragmentation occurred synchronously along the fragment (Additional File 5A) and, in others, advanced in a ‘saltatory’ fashion (Additional File 5B).

### Schwann cells regulate the number of axon fragments produced during Wallerian degeneration of the posterior lateral line nerve

**Figure 3:**
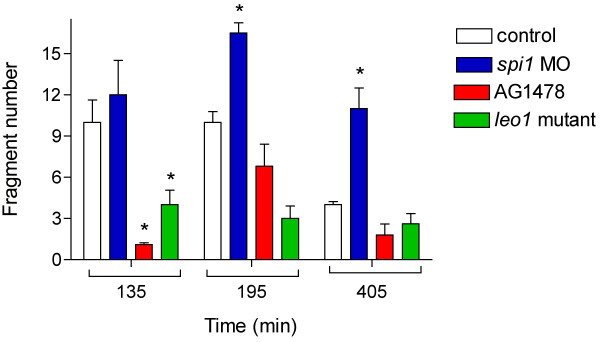
**Leukocytes participate in axon fragment removal.** Axonal fragments were quantified at three different time points with the Analyze Particle plugin of the ImageJ software (see Materials and Methods). At 135 minutes postaxotomy, larvae lacking Schwann cells (*leo1* mutants and larvae treated with the Schwann cell inhibitor AG1478) had significantly fewer axon fragments (*P* <0.05) than controls or *spi1* morphant larvae depleted of leukocytes. At 195 and 405 minutes *spi1* morphants had more axon fragments than controls or larvae lacking Schwann cells (*P* <0.05). n = 10 for each treatment; all experiments were carried out in *neuroD::GFP* transgenic fish.

Schwann cells and leukocytes are responsible for the phagocytosis of axon debris in many neuronal populations [11-13]. To determine whether these cells regulate pLL WD, we interfered with the development of each cell type separately and in combination (Figures 2 and 3). As in other peripheral nerves, Schwann cells surround the pLL nerve [41]. We inhibited development of Schwann cells using two strategies. First, we incubated fish in the ErbB inhibitor AG1478, which blocks the binding of neuregulin to the Erb receptor, effectively depleting peripheral nerves of Schwann cells [39]. Second, we used *leo1* mutant fish, which do not develop neural crest derivatives, including myelinating glia [42]. There was no difference in the timing of fragmentation or clearance between control fish and fish lacking Schwann cells, indicating that Schwann cells do not contribute to the timing of WD (Figure 2). However, fewer fragments were produced in these conditions with respect to the control group (Figure 3), indicating that Schwann cells may influence axon fragmentation after damage, but do not control the rate of WD. 

### Leukocytes regulate the onset of axon fragment clearance during Wallerian degeneration of the posterior lateral line nerve

**Figure 4:**
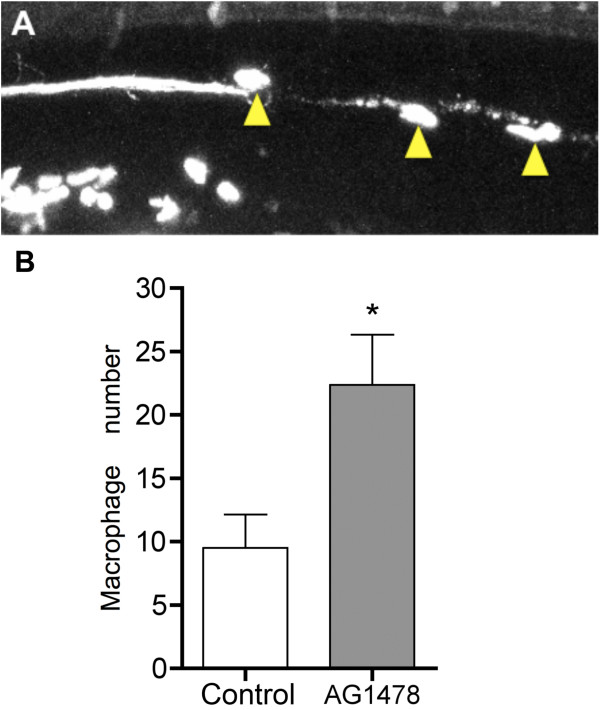
**Leukocyte migration to degenerating axons is enhanced in the absence of glia. (a)** Leukocytes (yellow arrowheads) homed to the degenerating pLL nerve in a *lysC::GFP*/*neuroD::GFP* double transgenic larva. **(b)** The number of leukocytes directly in contact with degenerating axons was significantly increased in the absence of glia (AG1478). Quantitative analysis was performed between 2.5 and 4.5 hours postaxotomy. Student’s *t* test, *P* = 0.00167.

Neutrophils and macrophages are professional phagocytic cells that play a role in the clearance of neuronal debris [43]. These cells also patrol the region near neuromasts and respond to injury in these sensory organs [44]. To determine whether immune cells are recruited to the vicinity of degenerating axons and whether this interaction is dependent on the presence of Schwann cells, we axotomized compound transgenic larvae harboring both the *lysC::GFP* transgene, which labels leukocytes [45], and *neuroD::EGFP,* which labels the pLL axons. Leukocytes migrated to the site of axotomy and appeared to interact with axon fragments. The presence of innate immune cells near the degenerating axon was augmented in the absence of myelinating glia (Figure 4). Thus, innate immune leukocytes present at this stage (mainly macrophages and neutrophils [45]) are likely to be important participants in the process of phagocytosis of axon fragment and debris clearance during WD. 

To test the hypothesis that leukocytes mediate phagocytosis, we used a morpholino targeting the *spi1* gene, which specifically ablates the myeloid lineage [46] (see Materials and Methods). Comparing WD kinetics in these fish and controls indicated that the timing of axon fragmentation was normal but that the clearance phase was significantly longer in immune cell-depleted fish (Figure 2), as axon fragments persisted for over six hours under these conditions (Figure 3). When we combined immune cell and Schwann cell depletion, the speed of clearance was restored to near control levels (Figure 2), indicating that cell types other than innate immune leukocytes can contribute to debris clearance, but only when Schwann cells are absent. Epidermal skin cells are strong candidates, since lateral line axons interact closely with skin cells in the absence of glia [35] and have been suggested as potential phagocytes of degenerating trigeminal axon fragments in zebrafish [4]. Double mutant fish lacking both leukocytes and Schwann cells did not survive long enough after axotomy for regeneration to be examined (data not shown).

### Posterior lateral line innervation does not influence survival, differentiation, or regeneration of hair cells

**Figure 5:**
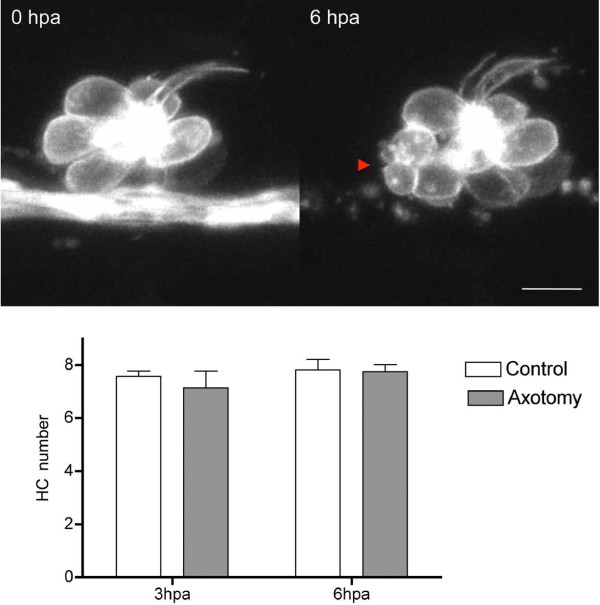
**Hair cells remain viable during Wallerian degeneration.** Top panel: A single neuromast of a three dpf double transgenic *neuroD::GFP/brn3c::GFP* larva expressing GFP in axons and hair cells was imaged for the first 10 hours after axotomy. At six hours postaxotomy, the nerve had degenerated and one hair cell had died (red arrowhead). Lower panel: total number of hair cells in the first neuromast at three and six hours postaxotomy (hpa); pLL degeneration did not significantly alter hair cell numbers. *Two-way ANOVA, *P* >0.05. Scale bar 50 μm.

Neuromasts and hair cells can differentiate in the absence of innervation, as has been observed in *neurogenin1* mutant and morphant fish, which lack pLL ganglion neurons [47,48]. To determine whether axotomy affects hair cell viability or the generation of new hair cells after neuromasts have differentiated, we imaged neuromasts in fish with axotomized pLL nerves. To accomplish this we used compound transgenic animals carrying the *brn3c::GFP* and *neuroD::EGFP* transgenes, labeling hair cells and pLL axons, respectively. These time-lapse movies showed that hair cells continued to differentiate, even as axons degenerated (Additional File 6). A few hair cells died during the 20 hours following axotomy, suggesting that innervation could influence hair cell survival (Figure 5 and Additional File 7). However, quantification of hair cell numbers in the first pLL neuromast of larvae fixed at three and six hours postaxotomy revealed no significant differences between axotomized fish and controls (Figure 5). Using the terminal deoxynucleotidyl transferase-mediated dUTP nick end labeling (TUNEL) assay to directly analyze cell death in axotomized fish, we observed labeling in neuromast cells surrounding hair cells, possibly corresponding to accessory cells of the neuromasts (data not shown). 

**Figure 6:**
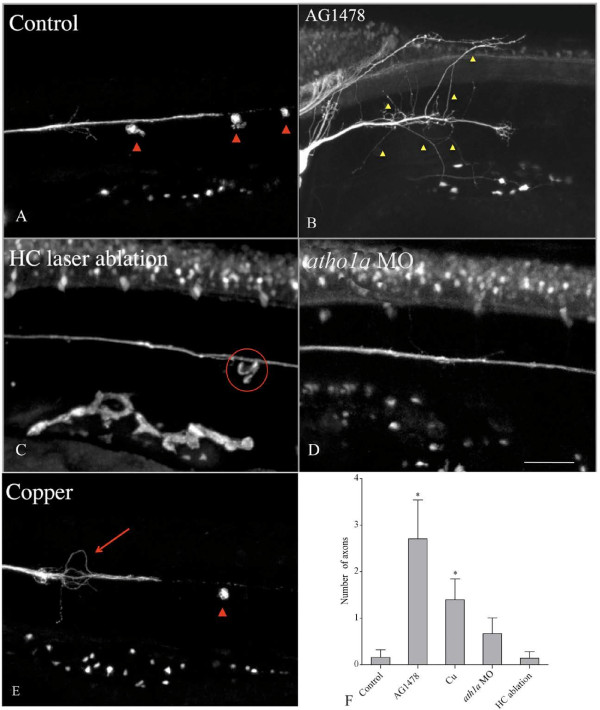
**Posterior lateral line (pLL) axon degeneration is affected by the absence of Schwann cells or hair cells.** Double transgenic larvae (*brn3c::GFP/neuroD::GFP*) were treated to remove Schwann cells (AG1478) or hair cells (laser ablation) prior to axotomy, and were examined after 24 hours to evaluate regeneration of the pLL nerve. **(a)** Control fish; red arrowheads indicate neuromasts; the nerve grew along its original path. **(b)** When Schwann cells were absent, axons regenerated after axotomy but many of them failed to grow along the normal trajectory (yellow arrowheads). **(c)** After physical ablation of hair cells in lateral line neuromasts (circle indicates position of ablated neuromast), axon regeneration was normal. **(d)** Axon regeneration was normal after inhibition of hair cell differentiation with the *ath1a* morpholino. **(e)** Ablation of neuromasts with CuSO_4_ treatment produced erratic growth of regenerating axons (arrow), but the normal trajectory of neurites was restored when neuromast hair cells (arrowhead) regenerated. **(f)** The number of axons wandering outside of the normal pLL trajectory was quantified and compared across the different conditions as indicated. *Significant differences between treatments (one-way nonparametric ANOVA, *P* <0.05).

Mammalian inner ear hair cells and pLL hair cells are structurally, genetically and functionally equivalent [49], but, unlike mammalian hair cells, pLL hair cells can regenerate after various toxic stimuli, such as damage by heavy metals [50-52] and antibiotics [53]. Our laboratory has standardized a protocol for using copper sulfate to ablate all LL hair cells, which can rapidly regenerate once copper is removed [50]. To determine whether regeneration of hair cells is perturbed in the absence of innervation, hair cells were ablated with copper sulfate and the axons of the pLL nerve in *neuroD::GFP* transgenic larvae were severed. Both the number of hair cells that reappeared and the timing of their reappearance were normal, indicating that innervation is not required for proper hair cell regeneration (see below; Figure 6E and Additional File 8). 

### Lateral line axons regenerate along their original trajectories

**Figure 7:**
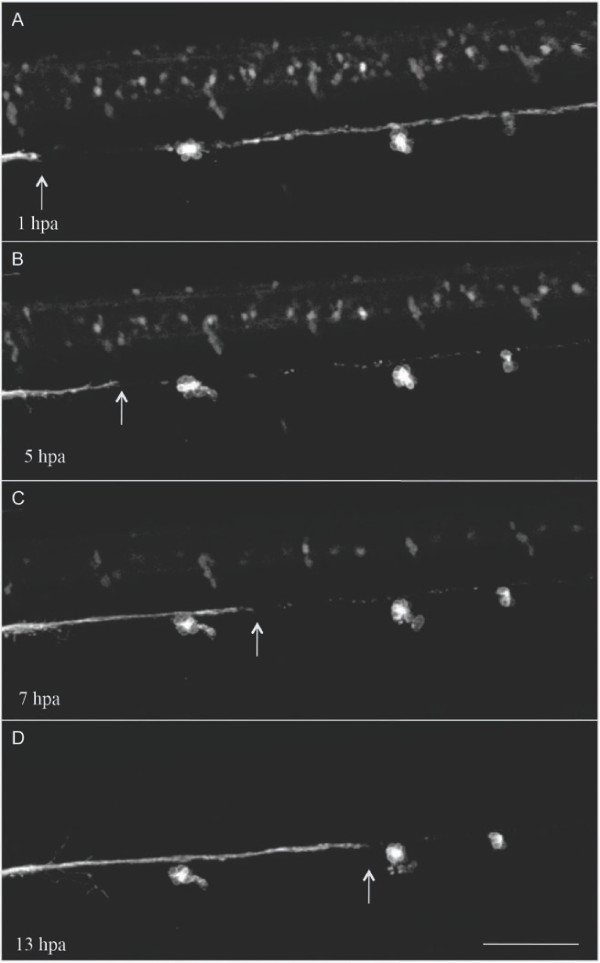
**Posterior lateral line axons regenerate after laser axotomy.** Representative images of the trunk of a three dpf *brn3c::GFP/neuroD::GFP* double transgenic larva imaged at the specified times (hours postaxotomy, hpa) near the lesion site. White arrow shows the leading axon tip. Scale bar, 100 μm.

In many species, axons in the peripheral nervous system can regenerate after axotomy [22]. However, few detailed studies of this phenomenon have been performed *in vivo*, prompting us to characterize axon regeneration in the pLL nerve. Neurites began to regrow from the proximal nerve a few hours after axotomy, but robust regeneration started when axonal debris from the detached fragment had cleared, approximately eight hours postaxotomy (Figure 7 and Additional File 9). The process was initiated by slowly growing pioneer neurites, and was followed by additional axons that closely followed the path of the first axon (Additional File 9). Axons regenerated at 0.3+/−0.1 μm/minute during the first phase of regeneration (up to six to eight hours postaxotomy) and then accelerated until a constant rate of approximately 0.8 μm/minute was achieved. By 24 hours after axotomy, the nerve had regenerated completely to the tip of the tail in all cases examined (n >20). The regenerated pLL was indistinguishable from the intact pLL prior to axotomy in terms of structure and trajectory. As fish survived the procedure without any visible defect or behavioral anomaly, the lateral line system appears to reacquire functionality after the pLL nerve regenerates. 

### Schwann cells are required for proper regeneration

The neuromasts, pLL neurons and Schwann cells that surround pLL axons develop in concert [27] and form a structurally and functionally integrated organ. In other systems PNS axon regeneration depends on the participation of various cellular components, including Schwann cells [23,26]. To test whether this is true in pLL regeneration, we eliminated Schwann cells in *neuroD::GFP* larvae with the ErbB inhibitor AG1478, and examined axonal regeneration over time. In the absence of Schwann cells, many regenerating axons grew in an erratic fashion, often diverting ventrally or dorsally from their path, a behavior never seen in control axons (Figure 6A, B and Additional File 10). Quantitative analysis of neurites diverging outside their normal growth pathway indicated a clear difference between regeneration in the absence of Schwann cells and controls (Figure 6F, n = 10). No significant differences were observed in the velocity of axonal growth between Schwann cell-deficient and control larvae (Additional File 11, n = 10). Similar results were obtained with *leo1* homozygous mutant fish (Additional File 12). These results suggest that the Schwann cells associated with the pLL nerve promote appropriate directionality during axon regeneration but do not affect the growth rate of regenerating axons.

### Axonal regeneration does not depend on intact target hair cells but is negatively affected by copper-induced ablation of neuromasts

pLL sensory axons innervate mechanosensory hair cells in neuromasts. To ask whether axonal regeneration requires target cells, we axotomized pLL axons and examined regeneration of the nerve in the absence of hair cells. In these experiments, we used double transgenic larvae (*brn3c::GFP/neuroD::GFP*) that label pLL axons and neuromast hair cells with GFP. To eliminate hair cells we used three different approaches: 1) laser ablation of all of the hair cells on one side of the animal prior to nerve axotomy. 2) injection of a morpholino that inhibits expression of *atonal homolog 1a* (*ath1a*), a proneural gene required for pLL hair cell development [54], 3) incubating animals in a high dose of copper sulfate (50 μM) for two hours [51], which eliminates neuromasts entirely (including hair cells and other cell types) (Figure 6C-E; Additional Files 13, 14 and 8). Neither laser ablation nor blocking hair cell differentiation with the *ath1a* morpholino altered the speed or trajectory of regenerating pLL axons compared with controls (Figure 6A, C-D; Additional File 11). However, regenerating pLL axons in animals lacking neuromasts as a result of copper toxicity displayed erratic behavior during the first phase of growth (eight out of fifteen animals). Regenerating axons initially projected dorsally and/or ventrally but eventually rejoined the correct path along the myoseptum, coincident with the appearance of new hair cells, which regenerated when copper was removed (Figure 6E; Additional File 8). Average velocity of neurite extension was similar between treated and control larvae (0.48 +/− 0.5 μm/minute for control and 0.51 +/− 0.3 μm/minute for copper-treated larvae), suggesting that the regenerative capacity of the axons themselves was not affected by copper exposure (Additional File 11). As the copper treatment eliminates not only hair cells but also other neuromast cell types [51], it is possible that correct pathfinding of pLL axons depends on cells other than hair cells. Alternatively, it is possible that the copper treatment directly caused the growing axons to regenerate aberrantly. To address this further, we eliminated hair cells with a fourth method: exposing animals to neomycin, a well-known ototoxic agent [53]. Although at three dpf this antibiotic does not kill all hair cells (approximately 40% of the pLL hair cells survived the treatment at this stage), a few axons showed a similar erratic behavior to that seen in copper-treated cells (1 ± 0.5 in treated vs. 0 in control larvae, n = 10; data not shown), supporting the notion that it is damage to neuromasts, rather than nonspecific copper toxicity, that causes the aberrant regeneration phenotype.

Finally, we examined nerve regeneration in embryos lacking both glia and neuromasts. Treating embryos with both copper and AG1478 strongly inhibited axonal regeneration (no regeneration observed after 20 hours, n = 9), suggesting that glia and neuromasts may have synergistic effects on regeneration of pLL axons, and emphasizing our conclusion that regeneration of pLL axons is strongly dependent on extrinsic cell types (Additional File 15).

## Discussion

To characterize the influence of nonautonomous tissues on axon degeneration and regeneration, we developed a reproducible method for severing the zebrafish pLL nerve *in vivo* and monitoring its behavior with time-lapse imaging. Imaging allowed us to visualize disconnected pLL axon fragments undergoing Wallerian degeneration and to monitor the entire process of axon regeneration, from injury to the reestablishment of pLL functionality. This system thus offers an unprecedented opportunity for analyzing the regulation of axon degeneration and regeneration *in vivo* and dissecting the roles of cellular and molecular regulators of these processes.

WD in the zebrafish pLL was extremely fast compared to axon degeneration in many previously characterized models. For example, the lag phase in the dorsal root ganglion nerve of the mouse lasts one to one and a half days after axotomy [7], a phenomenon that in the pLL of zebrafish larvae takes approximately three hours, similar to what was recently described for spinal motor neurons [55]. The elimination of axonal fragments took an average of around five hours in the pLL and twenty-four hours in spinal motor neurons [55]. Both of these phases are slightly but reproducibly faster in zebrafish trigeminal axons [4]. This observation indicates that WD in peripheral axons differ even in the same animal, suggesting either intrinsic or extrinsic differences between them. The fact that we were able to assess the success of WD just five to six hours after axotomy raises the possibility that medium-to-high throughput strategies for drug assays or genetic screens may be a feasible method for dissecting WD in this system. Imaging fragmentation along pLL axons (which can be longer than 1000 μm) demonstrated that WD proceeds from the distal end of an axon fragment to the proximal end, even though in a given section of a detached axon, fragmentation can appear to be synchronous. Surprisingly, this retrograde progression more closely resembles the outcome of a crush injury to the mouse sciatic nerve than the anterograde progression seen when the sciatic nerve is severed [56]. The signals mediating propagation along a severed axon fragment in one direction or the other are still unknown, but our results provide an additional model for investigating retrograde progression.

Zebrafish larvae can regenerate many tissues and structures [57], including the pLL nerve. By performing time-lapse imaging of regenerating axons, we observed new growth cones arising from the proximal axon stump immediately after degeneration of the distal fragment. In both pLL and trigeminal axons, regeneration starts only after debris from the original axon is cleared [58]. It is still not clear whether regeneration is dependent on fragment removal per se since, in trigeminal neurons, regeneration occurs on schedule when axon degeneration is delayed by Wld^S^ expression [4]. Nonetheless, these neurons seem to have evolved so that degeneration and regeneration occur in orderly succession.

Regeneration of the pLL nerve followed its original pathway, similar to what has been described for regeneration of zebrafish spinal motor neurons [55]. Analyzing regeneration of single axons labeled in transient transgenic larvae, we verified that reconstitution of a functional nerve corresponded to *bona fide* axon regeneration, rather than axon growth arising from new neurons. Regeneration of pLL axons qualitatively resembled axon regeneration described in invertebrates [59] and in some other vertebrate axons [60,61]. Regenerating pLL axons in wild-type animals grew at a speed of approximately 0.5 μm/minute, reaching all of the pLL neuromasts by 24 hours postaxotomy. These axons thus regenerated much faster than regenerating axons in the mouse central and peripheral nervous systems, where axons grow at 0.2 μm/minute and 0.07 μm/minute, respectively [7].

Axon fragments are known to be phagocytosed by at least two cell types: Schwann cells and immune cells. Ample evidence indicates that in the mouse PNS macrophages and glial cells jointly regulate the clearance of axons and myelin [13-16]. We found that in the absence of innate immune cells, pLL axon fragments persisted for significantly longer than in controls (Figure 2). Elimination of each cell type separately suggests that leukocytes are the main participants in the removal of axonal debris. Our findings are complementary to those of Rosenberg et al., whose work suggested that debris clearance after motor axon transection is mediated by macrophages, and that Schwann cells are dispensable for this process [55]. We also suggest that at least one other cell type must have the capacity to remove axonal debris because in the absence of both leukocytes and Schwann cells, axon fragments disappeared on schedule. Epidermal skin cells could fulfill this role as they associate closely with the pioneering pLL axons during their development and, in the absence of Schwann cells, pLL axons remain within the epidermis [36].

Interestingly, we found that Schwann cells are essential for proper navigation of the regenerating pLL nerve. In the absence of these cells, axons grew erratically, even though they extended at normal speeds. In the mammalian inner ear, several studies indicate that hair cells secrete factors that induce axon growth of acoustic ganglion neurons after injury (reviewed in [22,26]). Moreover, after Wallerian degeneration, the endoneurium that had surrounded the distal axon remains, leaving an endoneurial tube around which Schwann cells proliferate, forming bands of Büngner [61,62] that serve as a structural guide by which new axons navigate [26]. In addition to this structural role, after nerve injury Schwann cells express neurotrophic factors, which could contribute to axon guidance [63]. The erratic growth observed in regenerating pLL axons in the absence of Schwann cells, may thus be explained by the absence of a structural guide for regeneration or the lack of appropriate guidance molecules.

A recent report identified a fundamental role for glial cell line-derived growth factor (GDNF) in axonal guidance during development and regeneration of the pLL nerve [64]. In contrast with our results, that study found that Schwann cell absence did not affect regeneration of the pLL nerve, but that regeneration was dependent on GDNF production by interneuromastic cells. However, in that report, only a subset of Schwann cells were ablated (specifically, Schwann cells between neuromasts I and II). It is possible that some Schwann cells escaped ablation (for example, by dedifferentiating and losing GFP transgene expression) or that intact Schwann cells located beyond the second neuromast continued to exert an effect on the regenerating nerve.

Our study also addressed whether neuromast hair cells, the targets of innervation by the pLL nerve, are necessary for proper regeneration or pathfinding of axons in the pLL. Chemical elimination of neuromast cells (using copper toxicity) resulted in erratic growth of regenerating pLL axons, but axons reestablished the correct pathway upon regeneration of the neuromasts. This result suggests that the target cell environment influences the guidance of regenerating axons. However, when hair cells were eliminated genetically or physically with a laser, pLL regeneration trajectory and velocity were both normal, suggesting that hair cells themselves are not required for this process. A possible explanation for these contradictory results is that although copper treatment selectively damages neuromasts, without affecting surrounding glial cells, axons, or other tissues in the pLL, it can cause damage to multiple cell types in the neuromast, such as supporting cells and mantle cells, in addition to hair cells [50]. These other cell types in the neuromast were not affected by laser or genetic ablation. Thus, it is possible that the aberrant behavior of regenerating pLL neurites observed after copper treatment is due to neuromast accessory cell elimination. We cannot, however, exclude the possibility that copper may have an unexpected effect on the regenerating axons themselves or on the interstitial space through which they navigate. It will be important to use alternative methods of ablating neuromasts to further test their role in pLL axon regeneration.

While we did not find that innervation was required for the survival of most hair cells, it is possible that viability of these mechanosensory cells would be impaired after long-term denervation by afferent fibers, as is clearly the case in the mammalian inner ear [65]. We observed occasional hair cell death after axotomy, but most dying cells identified by TUNEL staining were located among accessory cells in denervated neuromasts. While it was known that pLL neuromast hair cells can develop and differentiate in the absence of a pLL nerve [47,48], this is the first study to examine their viability when denervation occurs postembryonically, after hair cells have developed and integrated normally into the lateral line. Nonetheless, reinnervation occurred within 24 hours after injury, due to the robust regeneration of the proximal axon, indicating that the hair cells are deprived of afferents for only a short period. It would be interesting to permanently block axonal regrowth and assess the survival of hair cells in the denervated state.

## Conclusions

The model we have developed exploits the high degree of conservation among the elements of the mechanosensory systems of vertebrates. The mechanisms of nerve degeneration, including WD, can be studied in a straightforward manner in the zebrafish pLL, offering a new tool to identify molecules and genes that regulate axon degeneration and regeneration. In this work, we have provided evidence for the roles of extrinsic cell types in the processes of lateral line axon degeneration and its subsequent regeneration, roles which are distinct and necessary for reestablishing a functional circuitry in this organ.

## Methods

### Zebrafish husbandry and genetic strains

Tübingen and AB wild-type, transgenic and mutant strains of zebrafish (*Danio rerio*) were maintained at 28.5°C on a 14-hour light/10-hour dark cycle [66]. The transgenic strains used were: *neuroD::EGFP* kindly provided by Dr. Alex Nechiporuk [67], *brn3c::mGFP* from Dr. Herwig Baier [68], *lysC::GFP* from Dr. Phil Crosier [45], *foxD3::GFP* from Dr. Darren Gilmour [27] and *mpeg1::mCherry* from Dr. Graham Lieschke [69]. We obtained the *leo1* mutant from Dr. Jau-Nian Chen [42].

All embryos were collected by natural spawning, staged according to Kimmel *et al*. (1995) [70], and raised at 28°C in E3 medium (5 mM NaCl, 0.17 mM KCl, 0.33 mM CaCl_2_, 0.3 mM MgSO_4_, and 0.1% methylene blue) in Petri dishes [71], unless otherwise noted. We express the larval ages in hours postfertilization (hpf) or days postfertilization (dpf). All experiments were carried out in 72 to 78 hpf larvae since, at this stage, the primary lateral line is completely developed and functional.

### Antisense morpholino and DNA injection

We used specific antisense morpholinos against the *atonal homolog 1a* (*ath1a*) and *Spleen focus forming virus (SFFV) proviral integration oncogene 1* (*spi1*) genes (Table 1) purchased from Gene Tools, Inc. (Philomath, OR, USA). Morpholinos (MOs) were injected into one-cell-stage embryos at the indicated concentrations. Experiments using control (mismatch and random sequence) morpholinos failed to elicit any discernible phenotypes and are not shown. We confirmed the efficacy of the *spi1* MO by injecting it into *mpeg1::mCherry* transgenic fish, which showed a nearly complete depletion of macrophages (Additional File 16).

**Table 1:** List of morpholinos used

**Targeted gene**	**Morpholino sequence**	**Injection volume**	**Morpholino concentration**
*ath1a*	5′-TCTGTTGGTTTGTGCTTTTGGGAGG-3′	8-10 nl	10 μM
*spi1*	5′-GATATACTGATACTCCATTGGTGGT-3′	1-2 nl	0.5 mM

Approximately 1 ng of *HuC::GFP* DNA (construct obtained from Dr. Hernán López-Schier) was injected into one-cell-stage embryos to visualize GFP in single lateral line neurons in mosaic larvae. GFP-expressing embryos were selected and axotomized as described below.

### Imaging and axotomy

pLL axons were imaged in stable transgenic *neuroD::GFP* zebrafish larvae and in transient transgenic *HuC::GFP* larvae. In most experiments, the entire nerve was severed in stable *neuroD::EGFP* transgenic larvae. A single labeled axon was cut in the case of transient *HuC::GFP* transgenic fish (for example, Figure 1 and Additional Files 1, 3, 4 and 5). Embryos were dechorionated, anaesthetized in 0.01% tricaine, and mounted in a sealed agarose chamber [34]. A Zeiss LSM510 confocal/two-photon microscope (Carl Zeiss AG, Oberkochen, Germany) was used to image and axotomize GFP-labeled lateral line neurons. Axons were visualized using a 25x water objective (NA 0.8), a laser wavelength setting of 910 nm, and 30 mW of power at the sample. To axotomize a specified region of the axon, a single 2D scan with 180 mW at a 70x ScanImage zoom was used. For time-lapse analysis, embryos were imaged at various intervals for one to twelve hours on a confocal microscope with a 20x, 0.5 NA air objective (Zeiss LSM 510). Approximately 15 optical sections were obtained at each time point, spaced 3 μm apart. These were compiled into Z-projections and movies with ImageJ and QuickTime software. Embryos were maintained at 28.5°C with a heated stage throughout imaging. Imaging for longer times or with older stages was not possible since after axotomy and treatments larvae became deformed, necrotic, or died. *mpeg1::cherry* transgenic larvae (Additional File 16) were imaged with an Olympus, MVX10 fluorescence dissecting scope (Olympus, Tokyo, Japan), equipped with a QImaging camera and Micropublisher 3.3, and were analyzed with ImageJ software.

### Drugs and copper treatment

A final concentration of 10 μM AG1478 (Calbiochem, Merck KGaA*,* Darmstadt*,* Germany) in 2% dimethyl sulfoxide (DMSO) was added to fish water beginning at 10 hpf until the end of the experiment to block ErbB signaling [72,73] and, thus, Schwann cell migration [41]. Confirmation of Schwann cell depletion from the pLL nerve was obtained by incubating transgenic *foxD3::GFP* transgenic fish in AG1478 and examining the larvae for the presence of labeled cells (see Additional File 17). For copper treatment, CuSO_4_ (Merck) was dissolved in E3 to a final concentration of 50 μM.

### Morphological analysis and data quantification

ImageJ software was used to quantify axonal fragments. The confocal images were binarized such that pixel intensity of regions corresponding to axons was converted to black and all other regions were converted to white [74]. Axonal fragments were quantified using the ImageJ Analyze Particles plugin, with the following settings: size pixel ^ 1 = 0-Infinity; circularity = 0–1; show outlines. The times obtained for different phases of WD were determined by blinded visual inspection of stacks and then averaged for each treatment.

### Statistics and analysis

We used one-way ANOVA for treatment comparison of parametric data or an equivalent nonparametric method (Kruskal-Wallis). Additionally a two-way ANOVA was used when the parameter depended on two factors (see text for details). The significance level was *P* <0.05 for all treatments. Values obtained from quantification of fragments were plotted against time. We used the Student’s *t* test for comparison of *spi1* morphants and controls. All data analysis was performed using Prism 5.0 (GraphPad Prism Software, Inc., San Diego, CA, USA).

## Abbreviation

AAD: Acute axonal degeneration; CNS: Central nervous system; DMSO: Dimethyl sulfoxide; DRG: Dorsal root ganglion; EGFP: Enhanced green fluorescent protein; GDNF: Glial cell line-derived growth factor; LL: Lateral line; MO: Morpholino; pLL: Posterior lateral line; PNS: Peripheral nervous system; TUNEL: Terminal deoxynucleotidyl transferase-mediated dUTP nick end labeling; WD: Wallerian degeneration.

## Misc

Rosario Villegas and Seanna M Martin contributed equally to this work.

## Competing interests

The authors declare they have no competing interests.

## Authors’ contributions

RV carried out most of the axotomy experiments and time-lapse imaging as well as quantitative and statistical analysis. SM carried out experiments where leukocyte development or Schwann cell migration were impaired, and imaged fluorescently labeled leukocytes and their interaction with degenerating axons. KO performed high temporal resolution time-lapse imaging of degenerating axons; SC carried out macrophage ablation experiments. RV, SM, KO and SC generated figures and additional files. AS and MA jointly wrote the manuscript, directed experiments and participated in the design of figures. All authors read and approved the final manuscript.

## Supplementary Material

Additional file 1 **Posterior lateral line axons degenerate and regenerate after transection.** Embryos were injected with the *HuC::GFP* transgene and a single labeled neuron was axotomized at 3 dpf. Larvae were imaged with a 20X objective every 20 minutes for 12 hours after laser axotomy. Representative time-lapse movie; each frame is a projection of a confocal image stack. Click here for file

Additional file 2 **Acute axonal degeneration occurs in the first two hours after axotomy.** Time-lapse movie after axotomy of the pLL nerve in a 3 dpf *neuroD::GFP* transgenic larva. Each frame is a projection of a confocal image stack; frames were captured every minute for 2 hours using a 20X air objective; field of view is 500 μM. Click here for file

Additional file 3 **Fragmentation proceeds in a distal-to-proximal direction.** Single axons of fish injected with *HuC::GFP* were transected at 72 hpf, and imaged every 2 minutes both adjacent to the axotomy site (proximal) and further down the length of the embryo (distal). A and B are images from two representative fish mounted laterally, with their left side visible. Fragmentation was observed (red arrows) in the distal segment earlier than in the proximal segment. Beading (white arrows), which precedes fragmentation, was also seen first in the distal segment. The length of time between the onset of fragmentation in distal and proximal segments is variable. Times shown are relative to the first image in each set. Scale bar, 100 μm. Click here for file

Additional file 4 **High temporal resolution movie of a single axon after pLL axotomy shows distal-to-proximal direction of degeneration.** Transient *HuC::GFP* transgenic larvae were axotomized at 72 hpf, and imaged every 2 minutes for 6 hours. Representative time-lapse movie using a 20X objective; field of view is 500 μM. Each frame is a projection of a confocal image stack. Click here for file

Additional file 5 **Within an axon segment, synchronicity of degeneration is variable.***A*. Fragmentation was synchronous within the span of axon segment shown. The axon segment was continuous at time 0 (left), and two minutes later was completely fragmented (right, arrows). Times are relative to the first image in each set. Fish is the same as that shown in Additional File 3A, distal. *B*. The axon segment shown underwent synchronous beading (white arrows, t = 2’), but fragmentation (red arrows) began at the distal end (t = 6’). Approximately 20 minutes elapsed before the fragmentation advanced towards the proximal end (left in these images), but progression of fragmentation along the remaining segment was rapid. In all images, right is more distal to the site of transection than left. Fish is the same as in Additional File 3B (distal). Scale bar, 50 μm. Click here for file

Additional file 6 **Hair cells survive and continue to differentiate during WD.** Double transgenic *neuroD::GFP* and *brn3c::GFP* fish were imaged every 20 min from 78 to 96 hpf using a 20X objective. Note differentiation of new hair cells even as the nerve was degenerating (arrowhead). Each frame is a projection of a confocal image stack. Click here for file

Additional file 7 **Neuromast hair cells persist during degeneration and regeneration of pLL axons.** Double transgenic *neuroD::GFP* and *brn3c::GFP* imaged with a 40X objective every 20 minutes between 78 and 96 hpf. During the entire sequence, only one hair cell died. Each frame is a projection of a confocal image stack. Click here for file

Additional file 8 **pLL nerve regeneration is temporarily impaired after copper-induced neuromast ablation.** Representative time-lapse movie of a double transgenic *neuroD::GFP* and *brn3c::GFP* larva imaged every 20 minutes from 78 to 96 hpf. Larvae were treated at 72 hpf with 50 μM copper sulfate, which kills most neuromast cells, and the pLL was axotomized. pLL axon growth was aberrant for the first few hours of regeneration, but fibers eventually reached the correct path along the myoseptum. Note that proper directionality of nerve growth was restored at the same time that neuromast hair cells began to appear by regeneration. 20X objective; each frame is a projection of a confocal image stack. Click here for file

Additional file 9 **The pLL nerve regenerates after transection.** Time-lapse movie of a double transgenic *neuroD::GFP* and *brn3c::GFP* larva imaged every 20 minutes with a 20X air objective. Each frame is a projection of a confocal image stack. Click here for file

Additional file 10 **Regenerating pLL axons grow erratically in the absence of Schwann cells.** Representative time-lapse movie of stable *neuroD::GFP* transgenic larva treated with AG1478 to inhibit Schwann cell development. Larva was imaged every 20 minutes from 78 to 96 hpf. Each frame is a projection of a confocal image stack. Click here for file

Additional file 11 **Depletion of Schwann cells, hair cells, or neuromasts does not alter the velocity of axon regeneration.** Average reinnervation velocity under different treatments showed no significant differences with control fish. Control, n = 10; AG1478 (Schwann cell absence), n = 9; *ath1a MO* (inhibited hair cell differentiation with the *atonal homolog 1a* morpholino), n = 10; HC ablation (all hair cells eliminated by laser ablation on the same side of the animal as axotomy), n = 8; Neo (elimination of hair cells by exposure to 200 μM neomycin), n = 10; Cu (elimination of neuromasts by exposure to 50 μM CuSO_4_), n = 15. Non-parametric analysis of variance, p <0.05. Click here for file

Additional file 12 **pLL axons exhibit erratic regeneration in *leo1 *mutant larvae after axotomy.** Representative time-lapse movie of a *leo1* homozygous mutant larva in the stable transgenic *neuroD::GFP* background. Larva was imaged every 20 minutes between 78 and 96 hpf with a 20X air objective. Click here for file

Additional file 13 **Laser ablation of hair cells does not inhibit pLL nerve regeneration.** Hair cells of double transgenic *neuroD::GFP* and *brn3c::GFP* larvae were ablated using a two-photon laser prior to laser axotomy at 78 hpf. Larvae were imaged with a 20X objective every 20 minutes from 78 to 96 hpf. Each frame is a projection of a confocal image stack. Click here for file

Additional file 14 **pLL nerve regeneration does not require hair cells.** Hair cell development was inhibited by injection of the *ath1a* morpholino at the one-cell stage in *neuroD::GFP* transgenic fish, and axotomy was carried out at 3 dpf. Image is a representative time-lapse movie demonstrating nerve regeneration between 78 and 96 hpf. Note that regeneration of pLL axons was normal despite absence of hair cells. 20X objective; each frame is a projection of a confocal image stack. Frames every 20 minutes. Click here for file

Additional file 15 **The pLL nerve fails to regenerate after axotomy in the absence of both Schwann cells and neuromasts.** Representative time-lapse movie from double transgenic *neuroD::GFP* and *brn3c::GFP* larva lacking both glia (AG1478 treatment) and neuromasts (copper treatment). Larva was imaged with a 20X objective every 20 minutes from 78 to 96 hpf. Note the failure of the nerve to regenerate. Each frame is a projection of a confocal image stack. Click here for file

Additional file 16 **S*pi1 *morphants lack macrophages.** The *mpeg1:mCherry* transgenic line expresses cherry fluorescent protein in macrophages. (A) A 54 hpf control transgenic larva shows mCherry expression in macrophages distributed throughout the body. (B) Transgenic *spi1* morphants completely lacked macrophages. At 72 hpf the morphant phenotype persists (data not shown). Click here for file

Additional file 17 **Treatment with AG1478 results in depletion of pLL Schwann cells.***foxd3::GFP* transgenic fish express GFP in neural crest derivates, including pLL Schwann cells. (A) A 3dpf control larva shows GFP-labeled Schwann cells surrounding the pLL nerve (bracket). (B) Representative image of a 3 dpf larva treated from 10hpf to 3dpf with the ErbB inhibitor AG1478. Treated larvae lacked Schwann cells (bracket demarcates the same region indicated in A). Click here for file
