# Supplementary figures and images for: Dynamics of degeneration and regeneration in developing zebrafish peripheral axons reveals a requirement for extrinsic cell types
Source: Neural Dev. 2012 Jun 8;7:19. doi: 10.1186/1749-8104-7-19 (PMC3780720; doi:10.1186/1749-8104-7-19)

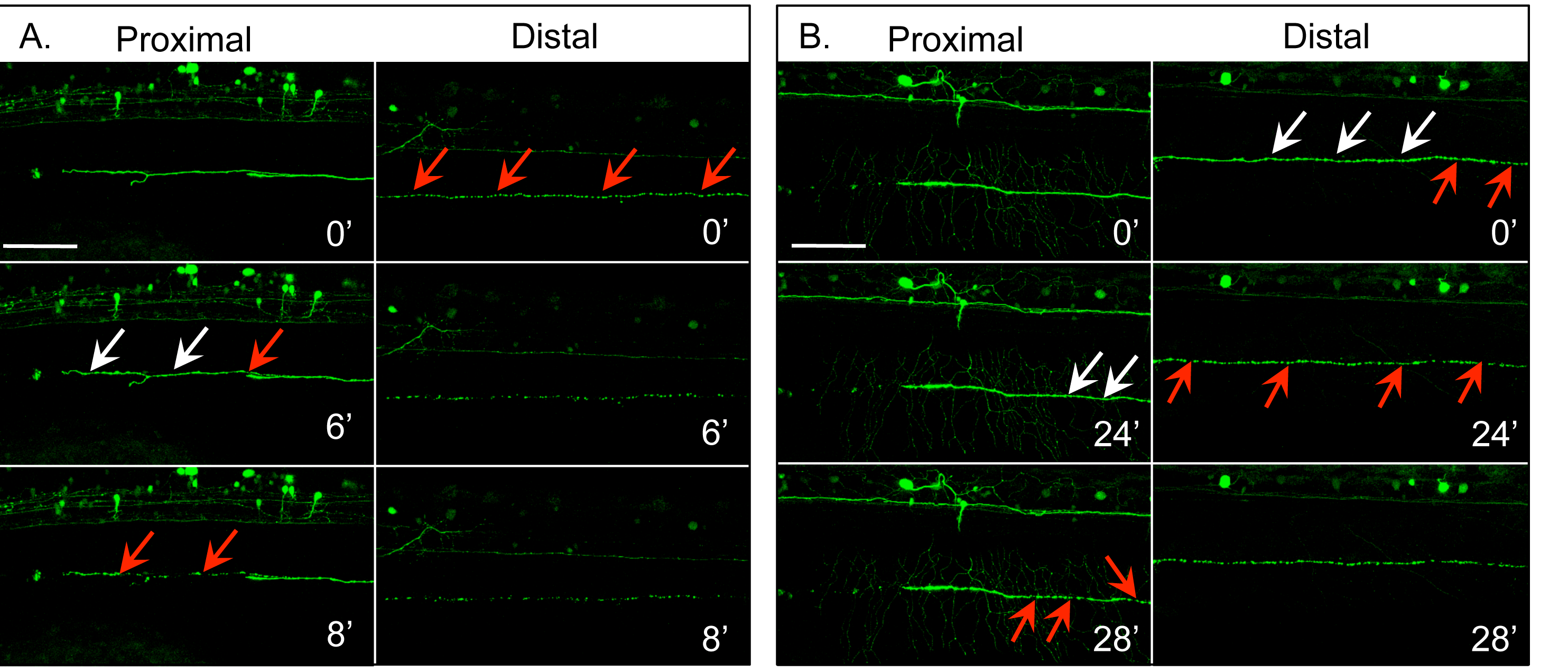

Supplement: Additional file 3 — Fragmentation proceeds in a distal-to-proximal direction. Single axons of fish injected with HuC::GFP were transected at 72 hpf, and imaged every 2 minutes both adjacent to the axotomy site (proximal) and further down the length of the embryo (distal). A and B are images from two representative fish mounted laterally, with their left side visible. Fragmentation was observed (red arrows) in the distal segment earlier than in the proximal segment. Beading (white arrows), which precedes fragmentation, was also seen first in the distal segment. The length of time between the onset of fragmentation in distal and proximal segments is variable. Times shown are relative to the first image in each set. Scale bar, 100 μm. [file 1749-8104-7-19-S3.tiff]

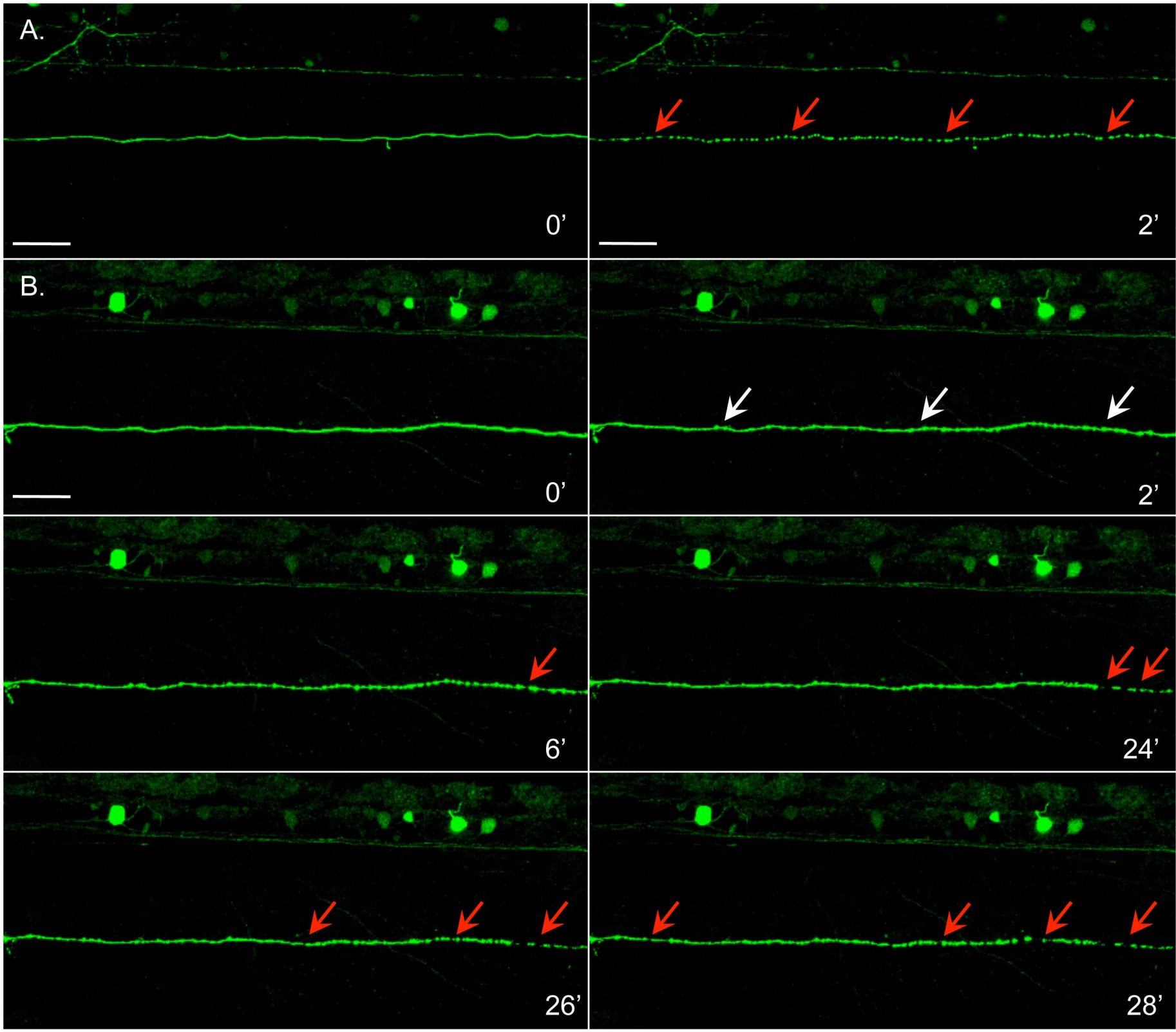

Supplement: Additional file 5 — Within an axon segment, synchronicity of degeneration is variable.A. Fragmentation was synchronous within the span of axon segment shown. The axon segment was continuous at time 0 (left), and two minutes later was completely fragmented (right, arrows). Times are relative to the first image in each set. Fish is the same as that shown in Additional File 3A, distal. B. The axon segment shown underwent synchronous beading (white arrows, t = 2’), but fragmentation (red arrows) began at the distal end (t = 6’). Approximately 20 minutes elapsed before the fragmentation advanced towards the proximal end (left in these images), but progression of fragmentation along the remaining segment was rapid. In all images, right is more distal to the site of transection than left. Fish is the same as in Additional File 3B (distal). Scale bar, 50 μm. [file 1749-8104-7-19-S5.pdf]

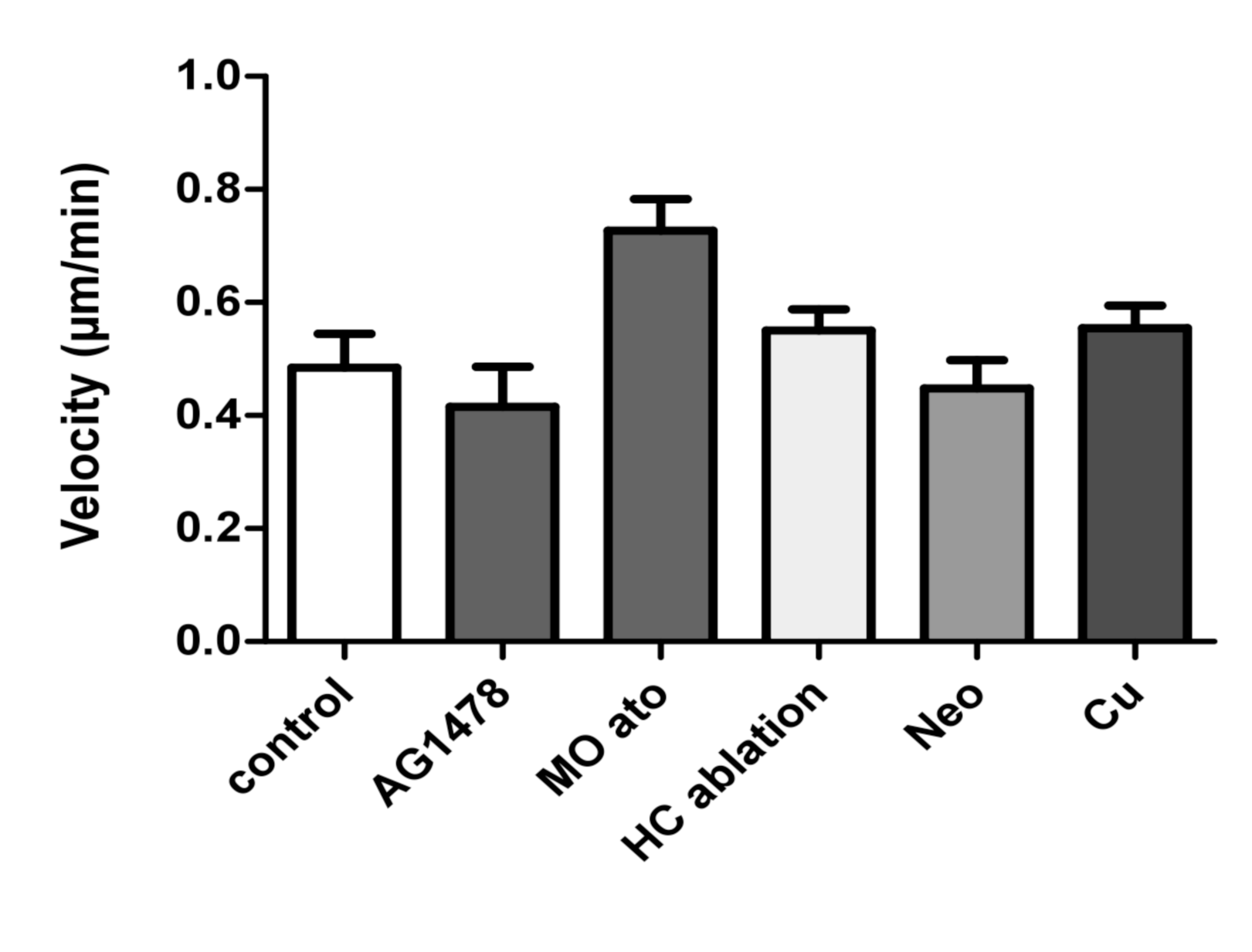

Supplement: Additional file 9 — The pLL nerve regenerates after transection. Time-lapse movie of a double transgenic neuroD::GFP and brn3c::GFP larva imaged every 20 minutes with a 20X air objective. Each frame is a projection of a confocal image stack. [file 1749-8104-7-19-S9.tiff]

A

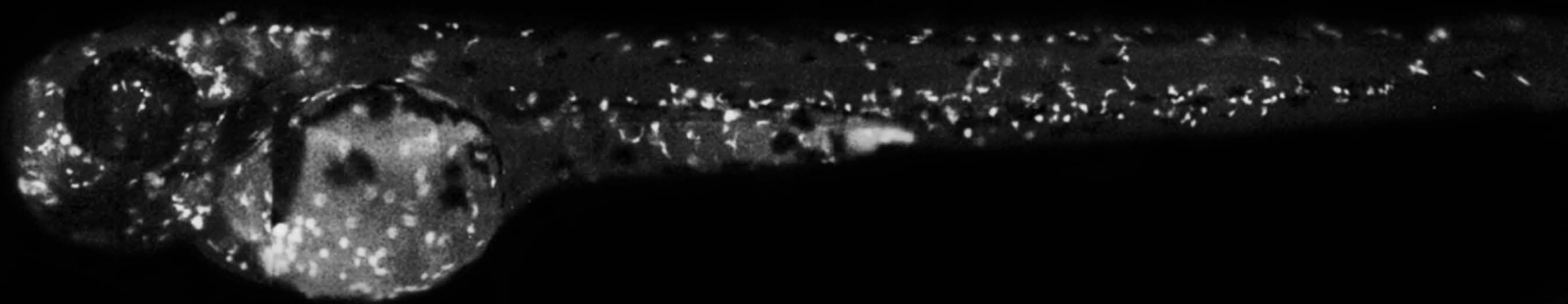

B

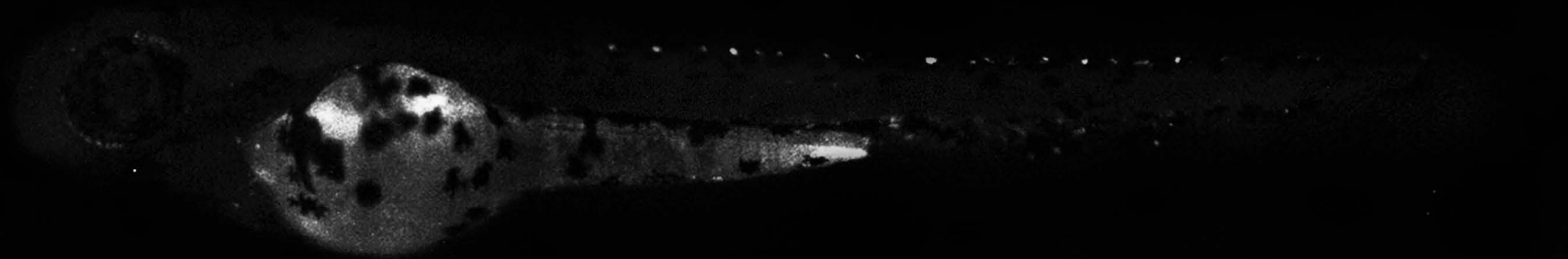

Supplement: Additional file 14 — pLL nerve regeneration does not require hair cells. Hair cell development was inhibited by injection of the ath1a morpholino at the one-cell stage in neuroD::GFP transgenic fish, and axotomy was carried out at 3 dpf. Image is a representative time-lapse movie demonstrating nerve regeneration between 78 and 96 hpf. Note that regeneration of pLL axons was normal despite absence of hair cells. 20X objective; each frame is a projection of a confocal image stack. Frames every 20 minutes. [file 1749-8104-7-19-S14.pdf]

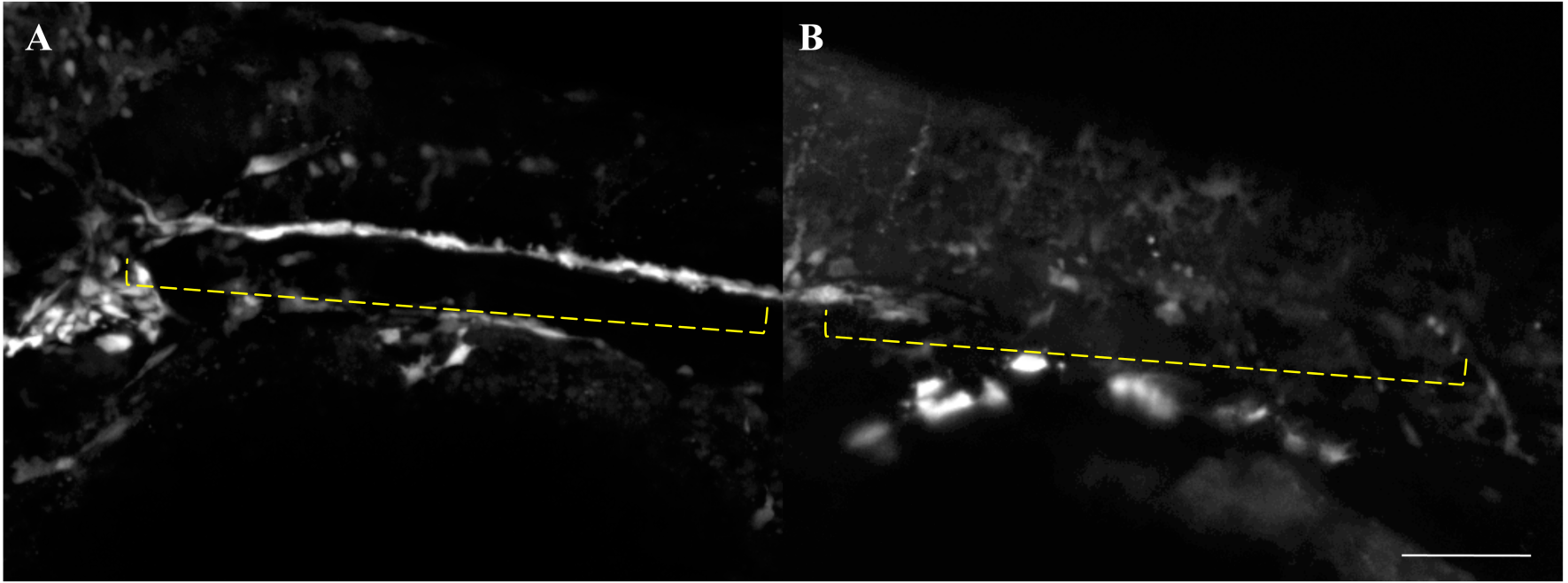

Supplement: Additional file 15 — The pLL nerve fails to regenerate after axotomy in the absence of both Schwann cells and neuromasts. Representative time-lapse movie from double transgenic neuroD::GFP and brn3c::GFP larva lacking both glia (AG1478 treatment) and neuromasts (copper treatment). Larva was imaged with a 20X objective every 20 minutes from 78 to 96 hpf. Note the failure of the nerve to regenerate. Each frame is a projection of a confocal image stack. [file 1749-8104-7-19-S15.tiff]
